# Eye Contact in Video Communication: Experiences of Co-creating Relationships

**DOI:** 10.3389/fpsyg.2022.852692

**Published:** 2022-04-25

**Authors:** Niclas Kaiser, Kimberly Henry, Hanna Eyjólfsdóttir

**Affiliations:** Department of Psychology, Umeå University, Umeå, Sweden

**Keywords:** eye contact, mutual gaze, social breathing, social interactions, perceptual crossing, participatory sensemaking, mediated conversation, online contact

## Abstract

As a result of the COVID-19 pandemic, increased number of persons have been forced to limit their interactions with friends and families to contact *via* video, which excludes eye-contact. The aim of this study was to examine individuals’ experiences of the difference between forced skewed visuality and the ability for eye-contact in conversations. Two custom-made units allowed 15 participants interacting in dyads to alternate between being able to make eye contact and having that ability removed through skewed visuality. Participants reported their experiences in semi-structured interviews. Data analyzed with qualitative content analysis resulted in three themes: *Shared eye contact allows us to create our relationship together*; *With eye contact, we adjust to each other to feel more connected and less intimidated*; and *We get more self-conscious when the visuality is skewed or shifting.* The results imply that skewed visuality as forced lack of eye-contact in video conversations effects embodied non-verbal processes related to sense of connectedness and participatory sensemaking, creating a sense of both emotional and physical distance, as well as heightening self-awareness about the need of actively regulating the other. We argue that this is one of the ways to understand the impact of moving interactions to online communication.

## Introduction

As a result of the COVID-19 pandemic, increased number of persons have been forced to limit their physical interactions with friends and families. Thanks to the widespread use of video-communication platforms, a lot of interaction involved seeing each other in video-calls, a technique that is specific in that it excludes the possibility for shared eye-contact.

The eyes, part of our enactive system, work with the brain, body, face, and voice in both neurological functions and social cognition (Gallagher et al., 2013). Studies of eye contact in social cognition have examined how the direction of one’s gaze can influence others and how eye contact during an interaction can influence both parties.

Gaze is a primary source of information in social encounters (Hirsch et al., 2017), as it indicates the mental and emotional states of others: a direct stare or averted gaze may signal aggression or submission, respectively, allowing us to predict our counterpart’s behavior and calculate our response (Emery, 2000). We also use gaze as a substitute for pointing to direct another person’s attention to something (Emery, 2000) to show intentionality, curiosity, warning, or the direction of an action. Gaze direction has also been shown to influence others’ interpretations of a person’s facial expressions of emotion; for example, a person whose gaze is direct while displaying active emotions such as anger or happiness is usually interpreted as being motivated to approach the observer (Adams and Kleck, 2005).

Gaze has a powerful ability to regulate interactions, express intimacy, and provide information about liking and attention (Kleinke, 1986). For example, an averted gaze or a glance at a mobile phone can indicate a wish to end an interaction, while a direct gaze can indicate interest in both the topic and the person talking. Individuals have been shown to react more positively to a direct, rather than averted, gaze, regardless of the emotion displayed by the other person (Hietanen, 2018). Thus, some people might prefer to be observed, even by someone who seems hostile, than to be ignored. People receiving high levels of direct gaze also perceive the observer to be more credible, likeable, and self-confident (Conty et al., 2016).

Direct gaze also has a self-referential effect in which the individual’s feeling of connection with those in the immediate context enhances prosocial behavior (Conty et al., 2016). Self-referral is related to people’s creation of positive attitudes about both themselves and those in the specific context of the immediate relationship, leading to a positive bias that motivates them to maintain the relationship (Leary, 2007). The self-referential effect can also enhance people’s ability to process memories of self-relevant information such as the faces and surroundings in a former interaction (Conty et al., 2016).

Unbalanced eye contact, however, when one party feels more directly observed by, than connected to the other, can also generate discomfort in some people. The direct gaze of another person, implying their focused attention, might enhance self-consciousness (Conty et al., 2016), which can lead to self-criticism and uncertainty about the observer’s intentions (Hietanen, 2018), and about their self-presentation (Hietanen et al., 2008).

While gaze by itself has an important relational function, it also influences other systems in an interactive relationship. For example, studies have demonstrated the ability to establish eye contact enhances mimicry (Wang et al., 2011), and mimicry enhances liking and affiliation between participants in an interaction (Lakin et al., 2003) even though it does not contain any new information, as opposed to the more engaged process of reciprocity. Mimicry improves harmony and flow in a conversation (Lakin et al., 2003), allowing it to continue without rifts or breaks, and also increases friendly and generous behaviors toward the mimicking person (Frith and Frith, 2008). However, this effect does not occur if individuals are aware of the mimicking and might instead be perceived as manipulative (Frith and Frith, 2008).

In summary, direct gaze and the ability to establish eye contact could have a function of information transfer, as well as affect the comfort of both parties and are therefore significant factors in social interactions. In this paper, we distinguish between the direction of *gaze* (direct or averted), in which the observer looks upon the observed, and *eye contact*, in which two individuals look into each other’s eyes.

### Social Interactions

Understanding the social roles of gaze and eye contact requires a basic understanding of social interaction as a biological co-regulative process for gaining the shared intentionality (Tomasello and Carpenter, 2007), participatory sensemaking (Fuchs and De Jaegher, 2009), and intersubjectivity (Stern, 2004; Schore, 2021) described as central to human sociality and the experiential dimension of connectedness. These concepts are argued to depend on an automatic, mutual, and time-sensitive process for engaging in the model of social breathing developed by Kaiser and Butler (2021). This model is based on the concept of whole brain–body interwovenness, closely connected to physiological entanglement (Palumbo et al., 2017) and interpersonal synchronicity (Koole and Tschacher, 2016). These terms are efforts to conceptualize a social process that is automatic, in the sense that it works partly precognitive and without the person being aware of engaging socially by will. And it is proposed that this engagement not only serves to engage in- and sustain the relational implicit process, but also that it is affecting both the relationship itself as well as the individual. This process should be understood as a neurologically underpinned process involved in allowing a person to develop implicit relational knowledge about oneself and the world by engaging with others (Kaiser and Butler, 2021). And because the eyes are such a key aspect in human sociality, they should also be considered in relation to the automatic social system.

Social interactions depend on: (1) co-regulation by the participants, making the interaction itself an autonomous process, and (2) the autonomy of both participants, making the interaction a dialogue with no one person solely dominating (De Jaegher et al., 2016). Merely observing others is fundamentally different to being in an interaction, which allows people to understand others’ minds through their responses and emotional engagement (Reddy, 2003; Redcay and Schilbach, 2019). In interaction, two people form a dyad with systemic properties (Hari, 2017), a self-organized social system that consists of more, and is greater than the sum of both individual’s intentions and characteristics. This system alone is a worthy subject of study (De Jaegher et al., 2016; Hari et al., 2016).

### Eye Contact in Social Interactions

The eyes have a perceptual function, through which people can perceive the perceptual activity of someone else’s eye (Lenay et al., 2011). When a dyad makes eye contact, each perceives the other perceiving them and their perceptual activity, creating the possibility for them to recognize, regulate, and influence each other in the interaction (Lenay et al., 2011). This meeting of perceptual activities, called perceptual crossing, is a key component of social relationships (Lenay et al., 2011) and has recently been shown to differ from just “being watched at” as arousal was enhanced only in perceptual crossing, when both participants could see each other (Jarick and Bencic, 2019).

People in a relational system can perceive and interpret a constant flow of non-verbal signals from each other such as gaze direction and mimicking to understand the other’s intentions, emotions, values, and beliefs and to successfully engage in the relationship (Frith and Frith, 2008; Kaiser and Butler, 2021). People can also signal, using gaze (Kleinke, 1986), facial expressions, body language, or non-verbal sounds when a social behavior needs to be regulated to avoid ruptures in the conversation (Roos et al., 2020). If a conversation is not perceived as synchronous, the social interaction will be unsuccessful and could cause a rift between the participants (Tomasello, 2014; Hari, 2017). And related to the eyes, dyadic pupillary synchrony has recently been shown to have a specific role in this in-and-out of synchrony process (Wohltjen and Wheatley, 2021), and arousal was also more enhanced from the direct gaze in co-located interactions than as seen in pictures (Hietanen et al., 2008; Pönkänen et al., 2011) or in video calls (Hietanen et al., 2020).

In summary, studies in the areas of eye contact and engagement in successful social interactions suggest that the individuals involved mutually regulate each other, with eye contact playing a notable part. Dyads might experience some social effects in mediated interactions such as video call, however, they cannot engage in simultaneous bi-directional eye contact on devices such as laptops and smartphones, and therefore cannot achieve visuo-perceptual crossing. We refer to this physically restrained ability to make eye contact as *skewed visuality*.

We argue not only that research into skewed visuality in interactions is limited, but also that there is an important difference between skewed visuality and differences in direction of gaze (averted vs. direct). The latter can disrupt one person in an interaction, while skewed visuality can disrupt the dyad’s intentional system as a whole. The aim of this study was to compare the experiences of skewed visuality versus having the possibility for eye-contact in individuals in dialog dyads.

## Materials and Methods

### Participants

Since the aim of the test was to examine social interactions with visual and auditorial stimuli, selected participant all had self-reported neurotypical social ability and normal or corrected-to-normal vision. The aim of the recursive selection process was to find dyads of close friends, romantic partners, or people sharing a living space who interacted with each other on a daily basis. This would allow the participants to remain minimally exposed to COVID-19 and would enable a relational flow in the interaction.

The collected data are based on 15 individuals (9 men, 6 women). Seven relationship dyads were composed of friends or partners, and the remaining participant conversed with one of the researchers. Participants were aged 23–48 years (*M* = 27), lived in different parts of Sweden, and had a similar range of experience with video calls. Since the relationship dyads consisted of friends or partners, they already liked each other and were comfortable interacting with each other. Participants were not financially compensated for their time or expenses such as transportation costs.

### Materials and Instruments

**FIGURE 1:**
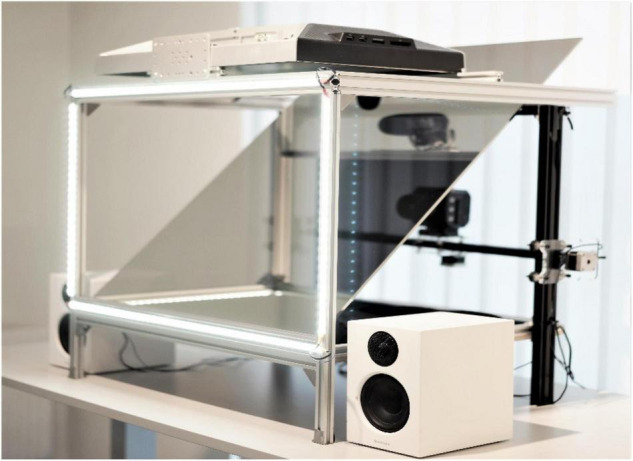
The NUNA setup.

**FIGURE 2:**
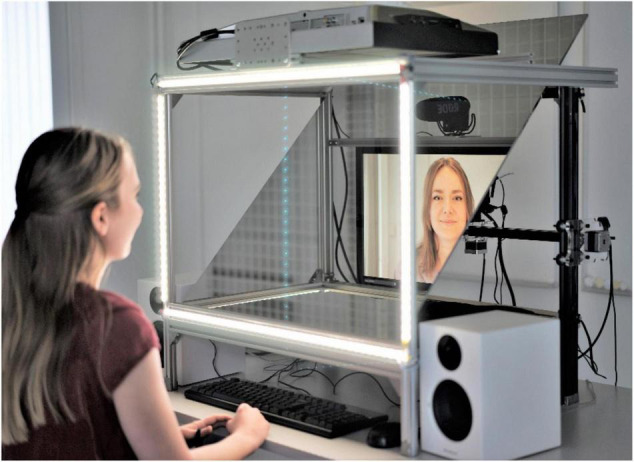
Demonstration of a conversation conducted in the NUNA.

Two identical setups (NUNAs) were built for the study. A NUNA (i.e., face in old Swedish) is a stand-alone communication unit intended to generate a video call situation in which camera angles can be manipulated to allow users to perceive shared eye contact. The NUNAs each had a teleprompter-style setup with a high-resolution camera (Panasonic BGH1) behind a 24- × 32-inch semi-transparent mirror set at a 45-degree angle reflecting a 24-inch LED screen; assignable LED lights; a set of speakers; and custom-built software (see Figures 1, 2). The camera had a robotized eye-follow function, allowing it to follow the gaze of the observer to the chosen part of the face on the screen.

The camera in the NUNA could alternate between an *offset* and a *centered* position. In the offset position, the camera was fixed above the screen, resulting in an offset of approximately 20 degrees meant to replicate the usual position of a web camera and to skew visuality and disallow eye contact. In the centered position, one of the NUNAs’ cameras was fixed at face-position between the eyes and the other followed the participant’s gaze, allowing participants to move in front of the NUNA and still be able to establish eye contact. The camera positions were controlled through external laptops.

### Procedure

Recruitment started with an informational text outlining the purpose and design of the study, the inclusion criteria, and the authors’ contact information. This text was sent to the authors’ personal networks and those interested were asked to contact the authors. This resulted in 11 participants who were asked to share the information with their own networks. Four additional participants were recruited *via* chain referral for a total of 15. After agreeing to participate, the dyads and the single respondent received an information sheet including a list of available test dates.

Upon arrival at the test location, the Umeå Institute of Design, the relationship dyads were accompanied by a test leader into two separate large rooms. The participants received written information about the test and a list of suggested holidays. They were instructed to individually rate how much they wanted to go on each holiday on a scale from 1 (Not at all) to 5 (Very much), a method used by Koudenburg et al. (2013) to prompt an effortless flow of conversation between the participants.

Next, the participants were placed in front of the NUNAs, approximately one meter from the cameras. The participants were then instructed to talk about the holidays with their partner for 16 min (participant no 15 did the conversation with one of the researchers). The camera began in the offset position usual in video calls and alternated between centered and offset every 4 min.

After the conversations, the authors conducted semi-structured interviews with the participants using a guide with three main questions asking about (1) the participants’ thoughts about the overall experience, (2) their thoughts about the conversation itself, and (3) whether they experienced a difference between the two camera positions. Each main question was designed to allow follow-up questions for clarification and further detail. Interviews finished with a final question asking participants if there was anything else they wished to add. The interviews, conducted in a one-on-one setting at the Umeå Institute of Design, lasted 10–30 min and asked for no personal information. They were audio-recorded on mobile phones, transcribed in verbatim and anonymized. The combined transcript consisted of 60 pages. All interviews were conducted and administered in March 2021 and data gathering followed the Swedish Public Health Agency’s regulations and general guidelines to prevent COVID-19 infections.

### Qualitative Analyses

To identify content in the participants’ narratives, data from individual interviews were analyzed according to qualitative content analysis (Graneheim and Lundman, 2004). Initially, we read the whole transcript several times to get acquainted with the data. In the next step, we separately cleared the transcript of data not related to the aim of the study and condensed the transcript into shorter units without changing its central meaning. We then compared these condensed units, corrected them through discussion, and created meaning units on a manifest level, which we reread, removing those not considered relevant the research question. The next part of the procedure aimed to generate meaningful transferable themes, corresponding to the participants’ narratives. This process was conducted by step-by-step arranging the information at hand to form a more abstract class, but without taking too large abstracting steps and thereby risk losing contact with the more manifest level. The generated three classes should thereby not be seen as essentially distinct, rather as necessary in the validity-strengthening processes of creating a discussable thread from the analytically highest class down to the more context-dependent and manifest meaning unit. The terms used for classes in this report were, from lowest abstraction level to highest: (1) categories, (2) sub-themes, and (3) themes. To identify bias among team participants, this process was combined with triangular intra-team comparison and questioning. Practically, the analytical process was conducted by looking for meaningful similarities and differences in the information at hand. And while sorting the information, the meaningful abstraction was preliminary titled in a way that the title included the content, but on a slightly more abstract level. Concretely, the remaining 375 meaning units were arranged into categories and preliminary sub-themes, resulting in 33 categories and four preliminary sub-themes which were later sorted into themes. No units at any level were erased during the analysis, only rearranged or renamed. In total, we identified 9 sub-themes and 3 themes. An example of the analysis is presented in Table 1.

**TABLE 1:** Example of the data analysis.

Meaning unit	Category	Sub-theme	Theme
Not looking at each other during a conversation feels wrong, I can feel it in my bones.	It feels natural to have eye contact in a conversation.		
		We establish an interpersonal interaction.	Shared eye contact allows us to create our relationship together.
I noticed very clearly that when we could share eye contact, we had a conversation; we were really talking.	With eye contact, it feels like we are united in the conversation.		

### Quote Editing

Quotations were altered to remove names and dialectal phrases that could link specific quotes to participants and translated from the original Swedish interviews into English. These changes to the quotes were made carefully to ensure the initial content was retained. “Double quotation marks” in block quotes and ‘single marks’ in in-text quotations were used to indicate quotation within quotations. Ellipses […] were used to indicate the removal of a phrase or sentence not relevant to the study’s aims.

### Ethical Considerations

The study was based on the four principles of humanistic and social science research of the Swedish Research Council (Vetenskapsrådet, 2002). Participants received written information about the study and the conditions of their participation, including their ability to discontinue at any time with no questions. Participants provided written informed consent as they arrived at the test location. Participants were also informed that use of the collected interview data would limited to the present study and that they were welcome to read the final product. The email addresses of the researchers were included in the information form to encourage participants to make contact if they wished or to ask any further questions. To protect the participants’ integrity, no names or identification numbers were used in the rest of the study. Because the study does not include themes that would require full ethical review in Sweden, the study was approved by a local committee at Umeå University.

## Results

The analysis resulted three themes and nine sub-themes (see Table 2), which are described in this section.

**TABLE 2:** List of themes and sub-themes.

Theme	Sub-theme
1. Eye contact allows us to create our	1(a). With eye contact, we seem to interact in the same space and time.
relationship together.	1(b). With eye contact, we establish an interpersonal connection.
	1(c). With eye contact, we are mutually engaged in the dialogue.
	1(d). With eye contact, we can share our intentions, goals, and actions.
2. With eye contact, we adjust to each other	2(a). We alternate our gaze during eye contact to avoid discomfort.
to feel more connected and less intimidated.	2(b). Our emotional needs benefit from eye contact.
	2(c). We use gestures as well as eye contact in our interaction.
3. We get more self-conscious when the visuality is	3(a). My discomfort with the level of eye contact changes my focus from the interaction to myself.
skewed or shifting.	3(b). I am responsible for improving a deficient interaction.

### Theme 1. Shared Eye Contact Allows Us to Create Our Relationship Together

This theme includes how participants connected to their partner in the NUNA when they could make eye contact. Participants described how the conversation relied on eye contact to establish a contextual connection based on presence, to have a dialogue rather than two monologs, and to share their intentions. When visuality was skewed, participants described feeling separate from their partners and not operating together.

#### 1(a). With Eye Contact, We Seem to Interact in the Same Space and Time

Participants described feeling physically close to their partner and having a co-located meeting when they could make eye contact in the centered position. In the off-set position, however, they reported feeling physically distant from their partner, some even felt lonely. Participants also perceived their partner as preoccupied and absent in the offset conversation and described feeling less connected and unsure whether their partner was paying attention to them and understanding them.

*I immediately noticed as soon as you looked away, and I understood that you were looking at something else on the screen, and how you then left the conversation. It was super noticeable, in a flash, and just. It was something I experienced deeply.*

Participants remarked that in the offset position they could give their partner the sense of eye contact by looking directly into the camera, but this meant they could not experience eye contact themselves, which they reported increased their awareness of being on a video call and emphasized the physical distance between them and their partner.

*[In the offset position] the person must actively turn and look into the camera to show that “I have eye contact with you,” but they never really share eye contact since they are breaking [their own eye contact]*. […] *I just think that it is odd.*

#### 1(b). With Eye Contact, We Establish an Interpersonal Interaction

Participants discussed the possibility that eye contact created a sense of togetherness. They said in the centered position they had a sense of mutuality, while in the offset position they felt detached from each other and the conversation felt unnatural and odd, which they related to the improbability of the offset angle happening in real life. With skewed visuality, participants reported a decrease in the interpersonal interaction, which they said was difficult to establish. Losing eye contact was described as losing the sense of relationship. The following quote illustrates how important eye contact was to people in the study.

*I got used to [the offset position] and we could have a conversation just fine, but it does not create the same magic as before.* […] *It creates something totally different when someone has eye contact with you and validates what you are saying just by holding your gaze. When it does not exist, it does not feel like you are validated* […] *I guess that is the magic.*

#### 1(c). With Eye Contact, We Are Mutually Engaged in the Dialogue

Participants said that the dialogue felt smooth and was easy to maintain when they could make eye contact. With eye contact, the dialogue felt satisfying and could flow naturally since they did not have to pay attention to seeking the gaze of their partner. In the centered position they could focus on the interaction, which they said the pair could look at each other as if they were co-located. The interaction in the offset position could feel somewhat comfortable and natural due to the dyads’ familiarity with each other, but participants still struggled to concentrate on their partner, causing the dialogue to feel disorganized and unbalanced.

*Our interaction seems a little bit “off” when it becomes, like* […] *“It is not like we are sitting and having a conversation, or it feels like you are below me.” Kind of like that. It moves the focus from the conversation and the essence of it, into thinking about where the other person currently is [on the screen].*

#### 1(d). With Eye Contact, We Can Share Our Intentions, Goals, and Actions

Participants described how eye contact helped them understand their partner’s social cues, including indications of whose turn it was to speak and whether what they were saying was interesting or boring, and to adjust the conversation accordingly. Participants said there being a risk of interrupting each other in the offset position since lack of eye contact made them unsure of their partner’s intentions and whether the conversation was heading in a shared direction. They also reported that since gaze was an important way of showing attention, an averted gaze signaled that their partner was more interested something other than the interaction.

*[I] get less contact with the other person since I cannot look into their eyes and read where the person is in their mind.* […] *It is easier to interrupt each other, and the turn-taking gets a little. Especially when you are not looking the person in the eyes either, then it is difficult to know; the turn-taking becomes extra difficult.*

### Theme 2. With Eye Contact, We Adjust to Each Other to Feel More Connected and Less Intimidated

This theme includes participants’ descriptions of how they regulated themselves and each other through eye contact and gestures in the conversation in the NUNA. Participants reported needing their partner to provide a desired level of eye contact and other non-verbal expressions in the conversation. Different levels of eye contact led to different sensations, with overlong exposure creating unwanted intimacy and no exposure needing to be compensated for with physical gestures. Alternating which partner initiated or broke eye contact, however, nourished participants’ needs for validation and made them feel seen without being stared at.

#### 2(a). We Alternate Our Gaze During Eye Contact to Avoid Discomfort

Participants described how the center position was enjoyable until they it seemed the eye contact had gone on too long, which was uncomfortable or unpleasant if it continued without the partner looking away or the NUNA changing camera angle. Participants said they expected their partner to occasionally avert their gaze to pause the conversation and contemplate. They compared a partner’s willingly fixated gaze to the feeling being stared at, which made them anxious. They perceived extended fixed eye contact as intense, overly intimate, and uncomfortable and responded by intermittently averting their own gaze.

*I feel very happy, the feelings are positive, but as I said before, it feels a bit. Well, I feel ashamed. It feels like “Wow, the other person really sees me now.” It is like they could be standing next to me right now. You cannot hide as much as you can in [the offset position]* […] *but [in the centered position] I felt almost naked in front of [my partner].*

#### 2(b). Our Emotional Needs Benefit From Eye Contact

Participants reported feeling validated by their partner in the centered position: they perceived their partner as warm, personal, and responsive and themselves as calm, relaxed, and comfortable. In contrast, they described their partner as cold, formal, and emotionally distant in the offset position. When eye contact was taken away, they felt frustrated, sad, and indifferent to the task, leaving them unmotivated and wanting to end the conversation. Participants explained that their emotions were closely related to making eye contact with their partner, which gave them a sense of validation.

*Frustration, irritation is what I felt. It bothered me when I said something and [the NUNA] kept moving and it changed into something else. It made me lose motivation to speak, it was not as enjoyable or exciting. Instead I felt dull feelings. I guess that is what I felt, frustrated and indifferent. [*…*] It was like “Well, what are we going to talk about now?” and I got bored and thought “Can we just end this?”*

#### 2(c). We Use Gestures as Well as Eye Contact in Our Interaction

Participants said the conversation in the NUNA resembled other video calls, explaining that their previous experiences influenced the conversation, so that in both camera positions the meeting could feel mediated and there was no change in flow. To resemble co-located meetings, mediated meetings required elements other than eye contact, such as a greater view of their partner, gestures, facial expressions, and body movements. Participants described the importance of these gestures in showing attention and participation in a mediated conversation.

*I notice if they are focused on my picture or something else, if they respond to my facial expressions or react to them, I believe that we mirror each other. [*…*] I can still see if his eyes are smiling [in off-set].*

### Theme 3. We Get More Self-Conscious When the Visuality Is Skewed or Shifting

This theme concerns the participants’ thoughts about themselves in the NUNA. Participants described how when their partner’s gaze was too fixed on them or was focused on something else, their attention shifted toward themselves. They noted that the shift between camera positions disrupted the conversation and made them feel self-conscious. They also felt self-conscious if they believed the conversation was flawed. This self-consciousness was reported to lead them to focus on their own words, appearance, and presentation to better respond to the current circumstances and repair the interrupted flow of the conversation.

#### 3(a). My Discomfort With the Level of Eye Contact Changes My Focus From the Interaction to Myself

Participants reported being very aware of themselves in the interaction, in both the off-set and centered positions. Their changed focus away from the conversation to themselves was related to how comfortable they felt, which was closely connected to the amount of time with eye contact and without eye contact. Participants expressed how in the offset position they were self-conscious and felt separate from, rather than part of, the interaction.

*I was self-conscious about how I spoke, that I was speaking incoherently and said strange things when we were not having eye contact [*…*] It feels like I am having a conversation with myself, I focus more on how I look.*

Participants also felt self-conscious in the centered position, since uninterrupted eye contact made the interaction feel intimate and awkward.

*I do not want to do it again. It was something very distressing in being that aware of the eye contact [*…*] I think that if there is something in between [the camera angles], that would have been nice. Not eye contact, like that, but not looking down.*

#### 3(b). I Am Responsible for Improving a Deficient Interaction

Participants reported that the centered position made them feel obliged to maintain eye contact to appear present in the conversation. They described how the centered position created pressure to focus on their partner and stay on topic. In the off-set position, however, they felt the need to do something, such as changing the camera position themselves, to achieve eye contact. Similarly, participants stated that the off-set position generated self-criticism related to them not performing well, including being unmotivated, rude, and unable to validate their partner.

*It felt hard because I noticed “Oh, I am not listening to anything [my conversation partner] is telling me right now, maybe I should be better at listening to [my partner].” It also made me feel frustrated with myself, like “Get it together! Listen!”*

## Discussion

The aim of this study was to compare how people in a conversation experience skewed visuality versus eye contact. The results, based on one-on-one interviews with 15 participants, were used to examine how skewed visuality affected the contextual relationship, the conversation, and the individuals in the NUNA.

The themes constructed in the result indicate how eye contact allows the partners to view the relationship as a unit working together in the interaction to have a functioning meeting. However, participants expressed heightened awareness of their own thoughts and visible actions in the NUNA when they felt there was too much or too little eye contact or when the camera shifted positions.

The following section discusses how the themes can be viewed through five prominent aspects in social theories: *Emotional distance*, *Non-verbal cues*, *Social breathing*, *Sense of physical separation*, and *Overcompensation*.

### Emotional Distance

A main finding in the participant interviews is the noticeable effects of skewed visuality in the interaction, especially in contrast to the occasionally opportunity to make and sustain eye contact. The participants said that the shift from the center to the offset position made them lose something in the interaction. Losing eye contact was described as uncomfortable and felt like a breach in the conversation. This can be explained in terms of people’s positive reactions to successful eye contact, which has been found to enhance prosocial behavior (Conty et al., 2016) and create positive attitudes about others through self-referral (Leary, 2007) contrasted with their less positive reactions to an averted gaze (Hietanen, 2018). These reactions were demonstrated when participants felt their partner was attentive and personal during eye contact and distant and formal when eye contact was not possible. As an averted gaze may indicate avoidance (Adams and Kleck, 2005), participants may have interpreted their partners as avoidant in the skewed visuality condition.

### Non-verbal Cues and Mimicking

The breach in the conversation when eye contact is difficult or impossible can also be related to an impaired ability to use and understand non-verbal cues to regulate each other’s social behavior (Frith and Frith, 2008; Roos et al., 2020). Although the participants could see each other’s gaze and gestures in skewed visuality, they still reported having difficulty interpretating those cues. If skewed visuality makes it difficult to read each other’s intentions and emotions through gestures, it might also be difficult to mimic these gestures, which in turn might reduce the flow in the conversation, since mimicry also affects conversational flow (Lakin et al., 2003).

### Low Social Breathing in Skewed Visuality

The narratives demonstrate how skewed visuality can be understood through the concept of perceptual crossing and participatory sensemaking. The NUNA facilitates perceptual crossing by allowing participants to make and perceive eye contact at the same time as each other. Perceptual crossing may have enabled the dyads to mutually recognize, regulate, and influence each other (Lenay et al., 2011), which might be why the participants experienced a separated, uninspired, and distressing interaction with skewed visuality. Another interpretation would be that as the eyes is a communicative modality for transfer of information, the reported experiences could be a result of restrained information transfer.

The descriptions of feeling divided from each other in the skewed visuality condition can also be understood through the social breathing model of Kaiser and Butler (2021). In the centered position allowing eye contact, the NUNA seemed to facilitate automatic and implicit processes of the dyad leading to high levels of social breathing, in which the dyad engages in embodied participatory sensemaking (Fuchs and De Jaegher, 2009) and gains a sense of “we-ness,” similar to findings showing specific embodied relational processes in online psychotherapy (García et al., 2021). In contrast, when the dyad had skewed visuality and expressed feelings of loneliness and detachment from each other, the interactions seemed to exhibit low levels of social breathing. Feeling lonely and detached in the conversation might also be related to low social breathing when difficulties arose in turn-taking and mutual understanding. Losing the sense of “we” can entail a loss of intersubjectivity, with the dyad no longer sharing their intentions, actions, and goals (Tomasello and Carpenter, 2007). A lack of shared intentionality might be what made our participants unable to adjust their behaviors to their partner’s. Without shared intentionality, individuals no longer have the same motivation of a shared agenda in the interaction and are expected, as in our study, to lose focus on the conversation.

### Physical Separation

In addition to feelings of psychological detachment, skewed visuality also seemed to bring a sense of physical separation that further hindered the dyads’ successful interactions. For example, in the centered position, eye contact allowed participants to feel as if they were standing in the same room, while they compared the skewed visuality in the offset position to being on a video call or even a phone call. The latter is an interesting comparison, since the camera was consistently active and participants could always see each other, unlike in normal phone calls, which further indicates how important ability to make eye contact became to the participants. Hietanen (2018) found that people were more strongly aroused in co-located interactions than in mediated video calls, and it has also been showed that heightened arousal is due to a process of going both ways (Jarick and Bencic, 2019). Although the participants’ physical arousal was not measured in the NUNA, their feeling as if the dyad shared physical space in the centered position suggests that they were aroused enough by the eye contact to compare the interaction to one in a co-located interaction. This might be yet another reason the participants felt such a difference when the NUNA shifted to skewed visuality.

### Overcompensation

Another finding was the participants’ experiences of how unregulated eye contact could become intimidating if it lasted for too long. While direct gaze can be a way of expressing intimacy (Kleinke, 1986), it can also signify a threat (Emery, 2000). Participants reported that extended eye contact led to feelings of both intimacy and intimidation, making them feel awkward and embarrassed. It is interesting to explore why the dyad experienced these struggles, even though they could have achieved perceptual crossing, and how they formed this intimidating impression of each other when the dyads consisted of close friends or partners who engaged in daily interactions. In their stories we find support for the explanation that the dyads’ unsuccessful attempts toward perceptual crossing in the skewed position made them eager to validate each other through eye contact, and that they therefore overcompensated when they had the opportunity. This interpretation is supported by participants’ descriptions of how urgently they wanted to take action to regain the connection and how pressured they felt to maintain eye contact to satisfy their partner’s needs.

The intimidation from prolonged eye contact might be understood as enhanced self-consciousness in the participants. Under a direct gaze, individuals have been shown to worry about how they present themselves (Hietanen et al., 2008) and might become self-critical (Hietanen, 2018), as our participants reported when eye contact continued longer than they were comfortable with. However, participants also stated that they felt self-conscious in skewed visuality, which might reflect their feelings of being in separate conversations and being inadequate conversation partners. This self-consciousness might have stayed with them when the NUNA changed camera angle and they had the possibility to share eye contact. When people are self-conscious, we can assume it interrupts their ability to engage in mutual regulation and maintain conversational flow, since the dyad functions as two separate individuals rather than a unit.

Yet another reason for these struggles might be that the novel opportunity to make eye contact in a mediated interaction was outside of their previous experience and left participant unsure of how to act or adjust to the situation. An adjustment from pre-reflective patterns onto reflective ones for sustaining interactive dynamics, just as reported in online psychotherapy (García et al., 2021), putting focus on the automatic relational embodied system (Kaiser and Butler, 2021).

## Conclusion

In summary, the present study put light on the eye’s meaning in several processes involved in natural social engagement. Although it has been showed that online interaction in, e.g., clinical settings is more than hindered contact (Downing, 2021; García et al., 2021) and that video based psychotherapy is highly effective (Cataldo et al., 2021), we find support for the notion that lack of eye contact effects the relational interactional process in a negative way. Both on lowering a sense of connectedness, creating a sense of distance, as well as heightening self-awareness about the need of actively regulating the other. A process that is discussed as automatic and pre-reflective social breathing for mutual embodied time-sensitive co-regulation for participatory sensemaking and intersubjectivity. This limitation is probably one of the ways to understand a sense of social isolation during a period of social physical distance when interactions are restrained to online communication.

## Future Research

Based on this study, we suggest that online video-based interaction and skewed visuality is a window for gaining further knowledge about the human social system and social connectedness. We propose interdisciplinary research focusing on the biological system underpinning these experiences in terms of right brain communication (Schore, 2021) physiological entanglement (Palumbo et al., 2017), participatory sensemaking (Fuchs and De Jaegher, 2009) and social breathing as full brain–body interwovenness (Kaiser and Butler, 2021) by the use of second-person neuroscience (Schilbach et al., 2013) and novel quantitative methods.

## Data Availability Statement

The raw data supporting the conclusions of this article will be made available by the authors, without undue reservation.

## Ethics Statement

Ethical review and approval were obtained from the local ethical committee. The study did not require a full application for the study on human participants in accordance with the national legislation and institutional requirements. The patients/participants provided their written informed consent to participate in this study. Written informed consent was obtained from the individual(s) for the publication of any identifiable images or data included in this article.

## Author Contributions

Theoretical framework and experimental setup were constructed by NK. Data collection and analyses were mainly performed by HE and KH with contribution and supervision from NK. HE and KH wrote the first draft of the manuscript which was continuously developed by all authors. All authors contributed to the study conception and design, read, and approved the final manuscript.

## Conflict of Interest

The authors declare that the research was conducted in the absence of any commercial or financial relationships that could be construed as a potential conflict of interest.

## Publisher’s Note

All claims expressed in this article are solely those of the authors and do not necessarily represent those of their affiliated organizations, or those of the publisher, the editors and the reviewers. Any product that may be evaluated in this article, or claim that may be made by its manufacturer, is not guaranteed or endorsed by the publisher.
